# The effectiveness of public health advertisements to promote health: a randomized-controlled trial on 794,000 participants

**DOI:** 10.1038/s41746-018-0031-7

**Published:** 2018-06-27

**Authors:** Elad Yom-Tov, Jinia Shembekar, Sarah Barclay, Peter Muennig

**Affiliations:** 1 Microsoft Research Israel, 13 Shenkar st., 46875 Herzeliya, Israel; 2 J. Walter Thompson, 466 Lexington Avenue, New York, NY 10017 USA; 3 Global Research Analytics for Population Health and the Department of Health Policy and Management, Mailman School of Public Health, Columbia University, 722 West 168th St., New York, NY 10032 USA

**Keywords:** Lifestyle modification, Computer science

## Abstract

As public health advertisements move online, it becomes possible to run inexpensive randomized-controlled trials (RCTs) thereof. Here we report the results of an online RCT to improve food choices and integrate exercise into daily activities of internet users. People searching for pre-specified terms were randomized to receive one of several professionally developed campaign advertisements or the “status quo” (ads that would otherwise have been served). For 1-month pre-intervention and post-intervention, their searches for health-promoting goods or services were recorded. Our results show that 48% of people who were exposed to the ads made future searches for weight loss information, compared with 32% of those in the control group—a 50% increase. The advertisements varied in efficacy. However, the effectiveness of the advertisements may be greatly improved by targeting individuals based on their lifestyle preferences and/or sociodemographic characteristics, which together explain 49% of the variation in response to the ads. These results demonstrate that online advertisements hold promise as a mechanism for changing population health behaviors. They also provide researchers powerful ways to measure and improve the effectiveness of online public health interventions. Finally, we show that corporations that use these sophisticated tools to promote unhealthy products can potentially be outbid and outmaneuvered.

## Online advertising: healthier ads promote healthier living

People who see specific health-promoting messages after searching online for weight-related terms are more likely to subsequently search for information on weight loss interventions. A team led by Elad Yom-Tov from Microsoft Research Israel in Herzeliya conducted a randomized trial involving 794,000 users of the Bing search engine who queried terms related to weight, diet, and exercise. Randomly chosen subjects were shown advertisements designed to promote healthy living, while all other users were shown standard ads. The researchers found that 48% of people exposed to the health-promoting advertisements made searches within the next month for weight loss information, compared with only 32% of those in the control group. The findings suggest that targeted online messaging can help change population health behaviors.

## Introduction

Hundreds of millions of dollars are spent on traditional public health advertisements annually.^1–7^ In theory, public health advertising can save money and lives by encouraging behaviors that prevent disease before it happens.^8^ While the objective of advertising investments (e.g., encouraging people to quit smoking) differs from those of private advertisers (encouraging people to purchase a good or service), the central idea is the same: to change behaviors.

Before online advertising, it was only possible to empirically test public health campaigns by randomizing small numbers of participants and to examine a few outcome measures.^1,2^ This makes it difficult to test to whom different forms of advertisement are best targeted.^3–6^

Humans vary greatly with respect to both their biology and their beliefs. Medical researchers use predictive analytics to mine databases of genetic information in order to target treatments to individuals who are more likely to respond to them. Similarly, private advertisers use predictive analytics to mine multiple sources of sociodemographic and behavioral data to better target individual consumers with the goal of changing their behavior. However, precision public health interventions have largely sat on the sidelines both due to the large sums of money required for targeted advertising and due to ethical concerns.

Ethical concerns arise for a number of reasons. First, participant data are collected without informed consent.^9^ Second, many in public health feel uncomfortable with the idea of manipulating individual behaviors, preferring instead to work with anonymous means to attempt to change behavior more generically.^10,11^ Such concerns have largely pre-empted the use of precision public health advertising, leaving only private firms to employ these tools.

In the private sector, Google, Microsoft, Facebook, and other internet-based companies provide online services for free in exchange for the information that drives precision advertising using “big data analytics”. Online ads targeted using data analytics can influence emotions and behaviors.^10,12,13^

First, advertisers can make educated guesses or small-scale tests about who might respond most to a given advertisement based on common search terms by topic. Then, advertisement can be randomized to be shown to users of search engines that search for such terms. Randomization provides a “gold standard” test of efficacy. Randomization can also provide causal information on how different sub-groups (e.g., young women) respond to an advertisement relative to others. Information on the experimental responses of different “architypes” of individuals can then re-tested with newer, more effective advertisements. This incremental approach—targeting, refining, and testing—has the power to produce online ads that affect beliefs and behaviors.

Big data companies—such as Facebook, Google, and Microsoft—conduct tens of thousands of randomized-controlled trials (RCTs) on their users every year.^14^ These results are invariably kept inside these companies, but the general process for evaluating advertisement efficacy is likely similar across companies.

Search advertisements are typically presented as textual advertisements that appear on a search results page coupled with a click through link to the advertiser’s site. More advanced versions include images in addition to (or instead of) the text. While it is rare that users click on ads, online advertisements have been shown to increase sales both for online display ads and in brick-and-mortar stores.^15^ Display and search ads are believed to produce similar impacts, and about 75% of this increase in traffic from the cited study was from those who never clicked through.

In this paper, we take the reader through this advertising process, and conduct, to our knowledge, the first fully randomized online public health communication’s campaign which tracks not only click response to ads, but also the searches made prior to and post advertisement display. Professionals from J. Walter Thompson (JWT), a leading advertising firm in New York City, developed a series of online ads aimed at improving exercise and eating behaviors among search engine users (“users”) who are overweight. We then experimentally tested these ads using a series of 10 RCTs, each for a different textual advertisement paired with unique click through content. Finally, we explored the impact of the ads on changing health behaviors as measured by future health-promotion searches.

## Results

### Descriptive analysis

During the month of the RCT, the experiment ads were shown 265,279 times and clicked 1024 times. Of these displays, the ads were shown 3108 times to 2996 users who could be tracked in their queries before and after. Additionally, during the month of the RCT, a total of 505,693 non-exposed users made at least one query such as the ones which triggered a campaign ad in the treatment population.

The majority of users were between the ages of 35 and 64, and females were more likely to see the ads than males (Supplemental Fig. 1). Of those over the age of 65, males and female users were about equal in number. A total of 36 tracked users clicked on the ads.

The tracked users and users who were not tracked were both successfully randomly assigned (Table 1).

**Table 1:** Percentage of users by age group and by gender among all people exposed to the ads and among the tracked population

	All users		Tracked users	
By age group	Control	Treatment	Control	Treatment
13–17	1.0	0.9	1.3	1.2
18–24	5.3	9.1	6.5	8.8
25–34	10.0	12.7	11.0	12.5
35–49	28.7	32.4	29.3	32.4
50–64	44.9	37.4	43.6	37.5
65+	10.1	7.5	8.4	7.6
By gender				
Male	31.0	30.8	26.9	30.4
Female	69.0	69.2	73.1	69.6

The 18-24 year olds are over-represented in the all user treatment population, while the 50-64 year olds are underrepresented in both tracked and all user population, p-values were <0.05 for age groups and gender

### Click through rate

The click rate is congruent with the click rate in other advertising campaigns.^16^ As shown in Supplemental Fig. 1, females were more likely to use terms which triggered the campaign ads, but there was a trend toward males having a higher click through.

### Exposure to textual ads and future target searches

A model predicting future target searches from prior interest in target searches and from exposure to campaign ads reaches an *R*^2^ or 0.314 (*p* < 10^−10^). As shown in Table 2, prior interest in target searches increases the likelihood of future target searches by 52% (slope = 0.52, standard error [SE] = 0.001; <10^−10^). However, exposure to campaign ads significantly increases the likelihood of future target searches by 15% (slope = 0.15, SE = 0.01; *p* < 10^−10^), especially in the absence of prior target searches. Stated differently, 48% of people who were exposed to the ads made future target searches, compared to 32% of the controls (a 50% increase). This difference is even greater when observing the population which did not have past target searches (30% vs. 15%).

**Table 2:** Model of the likelihood that a user will make future target searches

#	Parameter	Slope (SE)	*t*-stat	*p*-value
1	User made past target searches	0.525 (0.0007)	699	<10^−10^
2	User is in the treatment population?	0.149 (0.0106)	14	<10^−10^
3	Interaction of (1) and (2)	−0.202 (0.0143)	−14	<10^−10^

### Predictive analytics

We constructed a model to predict future target searches in the treatment population. Using only respondent characteristics (both behavioral and demographic) produced a model with an *R*^2^ of 0.414. When only previous query topics were added, the *R*^2^ was 0.410. When both were added, the *R*^2^ was 0.491.

### Cox hazards analyses

Table 3 shows the hazards ratios for the likelihood of future target searches for the sociodemographic and contextual characteristics of the ads. Recall that we correct *p*-values for the number of comparisons within each category. We discuss statistically significant results here. While the number of previous searches for keywords is associated with a very slight change in the HR for future keyword searches, the average person tends to make a large number of searches. None of the ads were particularly more effective than other ads in evoking a future keyword search. However, exposure to more than one ads increases the chance of a keyword search by 11% (HR = 1.11; 95% CI = 1.03, 1.20). Females were much less likely than males to perform a future search for keywords when exposed to an advertisement HR = 0.84 (95% CI = 0.76, 0.91).

**Table 3:** Cox proportionate hazards ratios (HR) and 95% confidence intervals (CI) associated with searcher characteristics

	HR	95% CI
Number of past searches by the user	1.0001^a^	[1.000082–1.000109]
Number of past target searches by the user	1.0011^a^	[1.000209–1.001951]
Number of past ads shown to the user	1.1146^a^	[1.025217–1.203941]
Hour of the day	1.0062	[0.999524–1.012954]
Title “Burn calories sitting”	0.9820	[0.888591–1.075501]
Title “Chores: The New Workout”	0.9362	[0.816441–1.055910]
Title “Drink Water Lose Pound”	1.0311	[0.926341–1.135788]
Title “Find a Hairy Partner”	0.9642	[0.806221–1.122249]
Title “Laugh Your Calories Off”	0.9754	[0.816440–1.134264]
Title “Lose weight watching TV”	0.9840	[0.832413–1.135643]
Title “Pimp Up Your Snack”	1.0125	[0.793617–1.231302]
Title “Your kids are an exercise”	0.9117	[0.727972–1.095497]
Title “'Swalty' Snacks are best”	0.9774	[0.797300–1.157544]
Page number	0.9561	[0.766738–1.145376]
Page position: Top	1.0094	[0.910639–1.108114]
Page position: Right	0.9014^a^	[0.823386–0.979331]
Page position rank	1.0191	[0.998109–1.040139]
Age group 18–24	1.0912	[0.919550–1.262805]
Age group 25–34	1.1736^a^	[1.012110–1.335038]
Age group 35–49	1.1160	[0.980089–1.251910]
Age group 50–64	1.0937	[0.957400–1.230037]
Age group 65+	1.1636	[0.985247–1.341941]
Gender: Female	0.8369^a^	[0.766715–0.906988]
Ad clicked?	1.0461	[0.682235–1.410057]

^a^Denotes variables statistically significant at *p* < 0.05. Variables with the prefix “title” refer to specific advertisements

## Discussion

We randomized Bing users to receive a professionally designed public health advertisement or to receive control (status quo) advertisements. We found that people who view online health promotion advertisements are much more likely to perform searches related to health promotion than those who were assigned “status quo” advertisements. The experimental effect sizes were large, with 48% of those with exposure to the text messages (and in some cases, the landing pages) more likely to perform future health-related searches while only 32% of the matched control group performed such searches.

At the population level, searching for specific health behaviors is associated with performing these behaviors in the physical world.^17^ For example, the number of people searching for information about cannabis is highly correlated with the known number of users of cannabis,^18^ the number of people searching for specific medicines corresponds to the number of prescriptions sold,^19^ and the distribution of birth events, as inferred from search queries, is extremely well aligned with the distribution provided by the Centers for Disease Control.^20^ We show that it is possible to alter the behavior of those with enough interest to conduct a search online, and show that it is possible to test such behavioral changes experimentally. With online advertisements, it is no longer necessary to stab in the dark with public health advertisement design. Nor is it necessary to guess who will respond to those advertisements. Rather, it is possible to systematically target users with advertisements to which they are most susceptible, thereby eliciting behavior change. Our identification strategies can, in theory, be used to continuously refine, randomize, and test the targeting algorithms on different user types. For instance, it is not only possible to target based on the users’ age, race, and location, but also on their characteristics as defined by their internet searches, shopping preferences, and even email content. The targeting algorithms can use the information to be “stepped up” until there is evidence that the user changes his or her behaviors.

Our study was susceptible to few limitations, including those typically inherent to RCTs. Perhaps the most important limitation is external validity since we ran the campaign on only one platform. Second, it is difficult to quantify the impact of the counterfactual advertisements that were shown to users. The counterfactual could be health promoting (e.g., gym memberships), neutral (e.g., vitamin supplements), or negative (e.g., unhealthy foods or products targeted toward high-risk groups). It is therefore possible that an ad with no efficacy could appear efficacious if the bulk of counterfactual advertisements discouraged future keyword searches.

Experimental offline advertisements have shown that it is possible to motivate health behavior change with traditional advertising modalities, such as associating behaviors with those of desirable social groups.^2^ The only published RCT of online health promotion advertisements we are aware of demonstrated that it is different audiences respond very differently to a given advertisement.^16^ For example, empowering advertisements were generally more effective at inducing future searches on smoking cessation than those that emphasized the negative health impacts of smoking. But this varied dramatically by the demographic characteristics of the viewers.

This also raises significant concerns for health departments and other agencies that could greatly benefit from access to the data underlying the health advertisements. Most large information technology firms provide services to users for free in exchange for access to user data. To best understand how to target users and change their behavior, it would be useful for them to have access to identified data that could be linked across multiple sources of big data. Like academic institutions, most public agencies require approvals that are difficult to obtain in part because institutional review boards have not adjusted to the nuances of big data research and partly because there is no clear opt in for users of online services. Clearly, cooperation between ethicists, big data companies, governmental bodies, and academia has great potential to advance population health. We show that it is not only technically possible to launch an online campaign that effectively improves health behaviors, but also that corporations that promote an unhealthy diet or a sedentary lifestyle can potentially be outbid or outmaneuvered.

## Methods

### Overview

Our RCT was conducted by Microsoft during April 2017 using the Bing Ads system. In this system, advertisers bid to place the ads when specific keywords are searched by users of the Bing search engine. Internet users of the Bing search engine who were logged into a Microsoft account and searching for pre-specified keywords were selected for this study. Eligible users were randomly exposed to JWT ads (treatment) or any other ads served up by the system (control). We then followed both the treatment and control users’ future search queries, and retrospectively examined past queries to build and interpret predictive models.

This study was approved by the Microsoft Institutional Review Board and was declared exempt by the Columbia University Institutional Review Board under the understanding that the Columbia University researchers would not have access to the data in any form other than the tabular results presented in this paper, and further that they would not seek funding for the study.

This trial was registered on February 2018 with ClinicalTrials.gov, registration number NCT03439553.

### User selection

Those who are motivated to change their behaviors are more likely to do so. As a result, advertisers often attempt to target individuals with some motivation to change. In this study, we attempt to improve the viewer’s diets and increase their levels of physical activity. The goal of the user selection process was therefore to identify individuals who were motivated for behavioral change due to social stigma or disease, and then present an advertisement that suggests a behavioral change that is within reach given their lifestyle. We therefore selected users who used search terms associated with social stigma or diseases related to poor diet or low levels of exercise.

### Randomization

The Bing Ads system is designed to randomize advertisements for experimental purposes. In this study, we selected users for inclusion if they (1) were using the Bing search engine; (2) logged into a Microsoft account; and (3) typed any of the following combination of terms:

1. (Weight, Overweight, Obesity, lose weight) AND (<none>, Hypertension, High cholesterol, High blood pressure, Exercise, Diabetes, bullying)
2. Hypercholesterolemia, Fat, BMI, Body fat, Big gut, Big and tall clothing, Easy exercises, Healthy diet, Easy workout, Plus size, Weight loss pill, Diet pill, Weight loss surgery, XXL

**Fig. 1:**
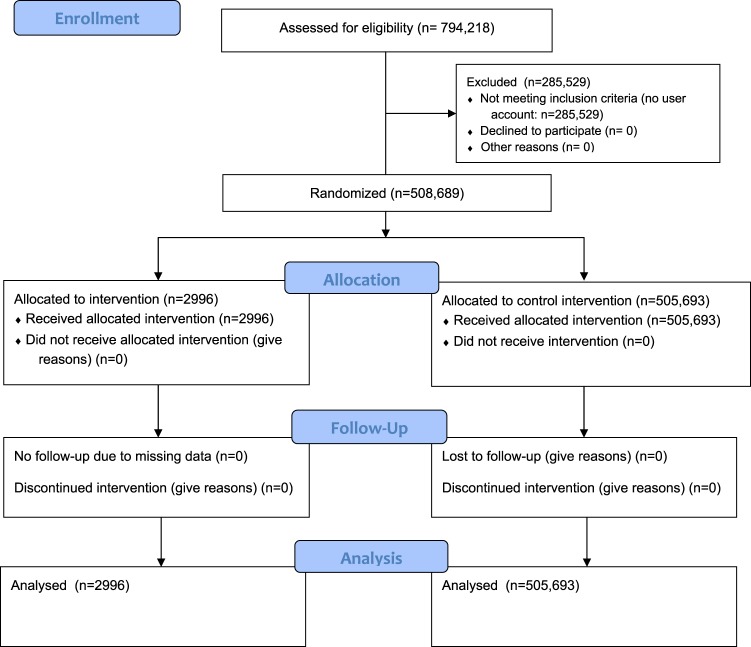
CONSORT 2010 flow diagram. CONSORT flow diagram template courtesy of http://www.consort-statement.org/consort-statement/flow-diagram

The vast majority of Bing search engine users who typed the above keywords (*n* = 283,716) were excluded based on a missing Microsoft account pre-randomization (the account is needed for user demographic data and analytics). Users who had a Microsoft account were further analyzed (Fig. 1). Among users with a Microsoft account, those with incomplete demographic data (age, gender, zip code) were also excluded, leaving 2996 treated participants and 505,693 control participants. The CONSORT diagram (Fig. 1) and the age and gender characteristics of the users (Table 1) show no threats to internal validity. There were no statistically significant differences in the demographic characteristics of the treatment and control users (*χ*^2^ test, *p* > 0.05).

In addition to the above four criteria for inclusion of users, campaign ads also had to competitively bid for an ad on the search results page. Keyword demand differs by advertisers, and so does the associated maximum bid for each keyword. To account for the differences in keyword demand and have a similar baseline for all keywords, we set the Bing Ads system to automatically adjust the bids for each of the campaign terms listed above to be high enough for our ads to be as competitive as control ads (i.e., those of other advertisers), but no more than US$1 per click.

### User characterization

We extracted all queries made on Bing by treatment and control users in our trial, from 1 month before the first advertisement was shown through until 1 month after the last ad was shown.

For each query, we registered an anonymized user identifier, the time of the query, the US county from which the user made the query, and the text of the query. The query was further classified (using a proprietary classifier developed by Microsoft) into one or more of approximately 60 topical categories. These categories were encompassed broad topics, such as commerce, travel, and health.

Users exposed to ads were further characterized by their self-reported age, gender, and the county-level poverty as inferred from the county from which they made the query.^21^

### Advertisements

JWT developed both the textual ads initially displayed to treatment users, as well as the content on the landing page shown when the user clicks on a textual link. The advertisements were grounded in the Fogg Behavior Model.^22,23^ In this model, three elements must come together at the same time: motivation to change, ability to change, and a trigger for change. The ads were designed to be “hot triggers,”^23^ designed to prime highly motivated users with content that is easy and actionable in order to nudge a behavior change toward more positive health habits.

All treatment users that enter the keywords above are exposed to the textual ads. The associated landing pages contained information on how the subject might improve health behaviors through nutrition or exercise. However, the vast majority of users will only view the textual advertisements. The JWT text ads fall into three categories: (1) suggestive of healthy behavior change (“Laugh your calories off”, “Chores: the new workout”, “Your kids are an exercise”, “'Swalty’ snacks are best”); (2) related to nutrition or exercise, but not to behavior change (“Burn calories sitting”, “Lose weight watching TV”, “Pimp up your snack”); or (3) unrelated to both behavior change and nutrition or exercise (“Find a hairy partner”). Each textual ad was designed to motivate users to click on the ad. Both the textual and click through advertisements were explicitly designed to avoid stigmatizing obesity.

The landing page ads focused solely on nudging users toward changing their behaviors with suggestions for incorporating small amounts of exercise or easy dietary changes into day-to-day activities. These were accompanied by an animated image meant to reinforce the message of the advertisement (see the Supplemental Appendix). Users were also provided links to additional content developed by professional health organizations or the Centers for Disease Control and Prevention if they wanted more information.

### Outcomes and predictor variables

Our primary outcome measure was the likelihood of a future search using a set of pre-specified keywords. These keywords were selected by identifying common weight-related search terms among Bing users. The terms fell into categories that suggest that the subject either (1) desires a deeper understanding of obesity (fat; nutrition; calories; body mass index; BMI; body weight; body mass) or (2) wishes to change their behavior (weight loss; weight watcher; weightwatcher; losing weight; and lose weight).

We were interested in exploring differences in outcome measures for treated and control users overall, by demographic characteristics, and by advertisement characteristics (content, placement, etc.). We were also interested in building predictive analytics that could identify which user types are most likely to respond to a given advertisement.

We used the following covariates to operationalize demographic and advertisement characteristics:

1. Past user behavior:
  - Number of past searches by the user
  - Number of past target searches by the user
  - Number of past ads shown to the user

2. User demographic:
  - Age group (categorized into six groups: 13–17, 18–24, 25–34, 35–49, 50–64, or 65+ years)
  - Gender (female or male)
3. Advertisement information:
  - Hour of the day ad shown (integer between 0 and 23 h)
  - Advertisement title (categorized into 10 groups)
  - Was the ad clicked? (yes/no)
  - Search page number on which the ad is displayed (integer between 1 and 100)
  - Search page position (indicator variable for whether the ad was placed on the top or the right-hand side of the search page)

### Statistical analysis

Given the large sample size, we specified an effect size of greater than or equal to 10% to be meaningful.

We explored the likelihood of future target searches given user’ exposure to ads, controlling for past searches. We used ordinary least squares regression to model the association between variables:

$$y = \alpha _0 + \alpha _1x_1 + \alpha _2x_2 + \ldots + + \alpha _Nx_N,$$

where *y* is an indicator of future searches, and *x* the predictors of the model.

Because previous searches predict the probability of subsequent searches, we were also interested in the interaction term between the probability of a subsequent search given exposure to the treatment (the interaction between the coefficient of conducting a previous search and being in the treatment group).

We then developed a predictive model. In this model, each user was profiled prior to the ad campaign with respect to demographic characteristics and previous topical searches. With respect to topical searches, we explored whether the user had performed searches that relate to one of 60 pre-specified categories of interest. These included broader topics, such as shopping, travel, and health. By adding a term to the above equation that includes previous searches in each of these categories, it becomes possible to examine the influence of inclusion of the topic on the model’s predictive value, as measured by the model’s goodness-of-fit (*R*^2^).

These models included all 10 of the covariates listed above. These covariates are used for predictive purposes, and regression is conducted on a cohort that has already been randomized. This way, it becomes possible to make predictions based on treatment response when only treatment status introduces non-random variation.

Next, we used Cox proportionate hazards models and explored 32 predictors of future searches:

$${\mathrm {HR}} = \exp \left( {X_1\beta _1 + \ldots + X_N\beta _N} \right),$$

where HR is the hazard ratio, *X* the predictors of the model, and *β* their corresponding model coefficients.

The predictors fell into five broad characteristics of the user and their exposures: previous searches, exposure to our advertisements, advertisement placement characteristics, age, gender, and poverty. We used Bonferonni correction for the number of categorical variables within each of these broader categories. We examined the HR for future searches for various user and ad characteristics.

In a secondary analysis (see Supplementary Materials), we used propensity score matching of users meeting inclusion characteristics, who were matched to unexposed users based on age, gender, and zip code, and analyzed using the above characteristics. This analysis allows for a low-noise, low sample size analysis in which it becomes possible to obtain very conservative assurances that there are statistically significant differences by treatment status, rather than relying on clinically meaningful effect sizes (as in the parent analysis).

### Data availability

The data that support the findings of this study are available from Microsoft, but restrictions apply to the availability of the data. Specifically, all aggregate advertising data are available from the authors on reasonable request. Individual-level search data are available from the authors on reasonable request and with permission of Microsoft.

## Electronic supplementary material

Supplementary Materials

## Electronic supplementary material

**Supplementary Information** accompanies the paper on the *npj Digital Medicine* website (10.1038/s41746-018-0031-7).
