# Author Correction: The effectiveness of public health advertisements to promote health: a randomized-controlled trial on 794,000 participants

**DOI:** 10.1038/s41746-018-0047-z

**Published:** 2018-08-16

**Authors:** Elad Yom-Tov, Jinia Shembekar, Sarah Barclay, Peter Muennig

**Affiliations:** 1 Microsoft Research Israel, 13 Shenkar st., 46875 Herzeliya, Israel; 2 J. Walter Thompson, 466 Lexington Avenue, New York, NY 10017 USA; 3 Global Research Analytics for Population Health and the Department of Health Policy and Management, Mailman School of Public Health, Columbia University, 722 West 168th St., New York, NY 10032 USA

**Correction to**: *npj Digital Medicine* 10.1038/s41746-018-0031-7, Published online: 27 June 2018

In the original version of the published Article, there was an error in the caption to Table 1 which stated “None of the differences are statistically significant (*χ*^2^, two-sided, *p* > 0.05)”. This has been changed to “The 18–24 year old are over-represented in the all user treatment population, while the 50–64 year old are underrepresented in both tracked and all user population, *p*-values were <0.05 for age groups and gender.” This has been corrected in the HTML and PDF version of the Article.

