# Supplementary material for: The effectiveness of public health advertisements to promote health: a randomized-controlled trial on 794,000 participants
Source: NPJ Digit Med. 2018 Jun 27;1:24. doi: 10.1038/s41746-018-0031-7 (PMC6550260; doi:10.1038/s41746-018-0031-7)
Supplement: Supplementary file 1 — Supplementary Materials [file 41746_2018_31_MOESM1_ESM.pdf]

Supplementary Materials

Figure S1 shows the percentage of impressions by gender and age group for all users who were exposed to the campaign ads. Females and people aged 35-64 were more likely to ask using keywords that triggered the campaign ads. Figure S2 shows the percentage of ads which were clicked by users (the click-through rate, CTR) by age group and gender. Here, males and people aged 35-64 were more likely to click the ads when shown to them.

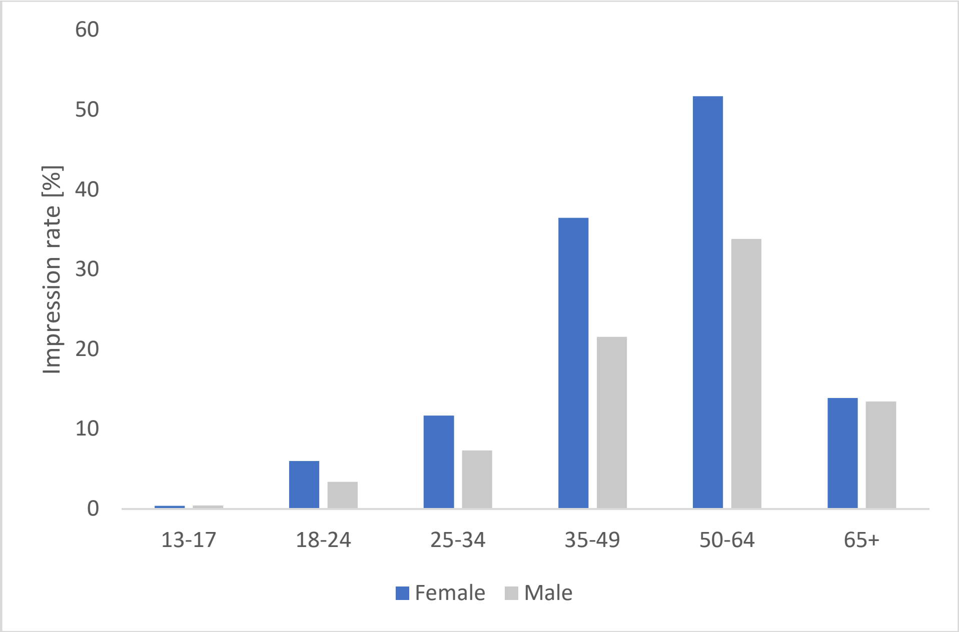

Figure S1: Percentage of ads shown to different genders and age groups

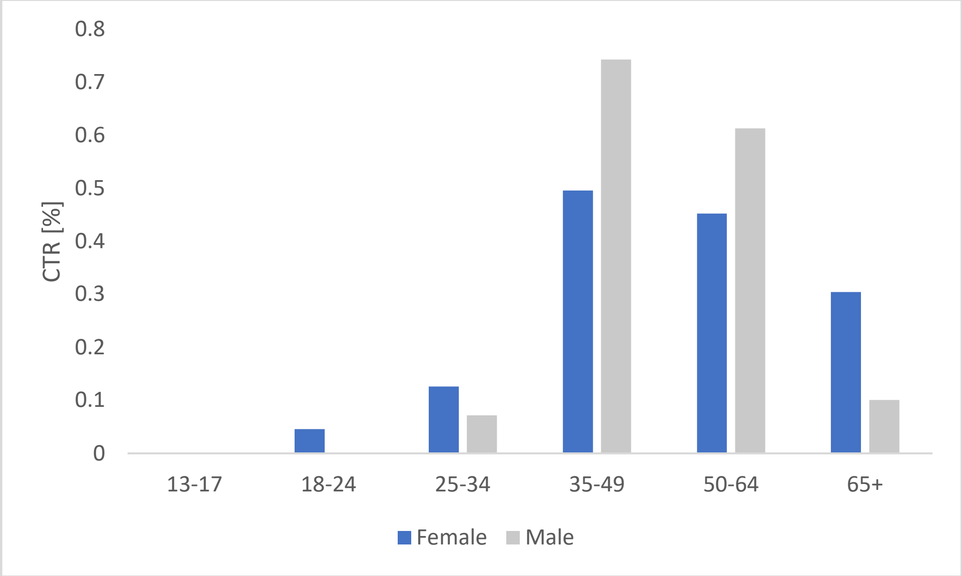

Figure S2: Click-through rate (CTR) by age group and gender.

Figure S3 shows the CTR for the different advertisements. As the figure shows, some ads were much more likely to attract clicks than others.

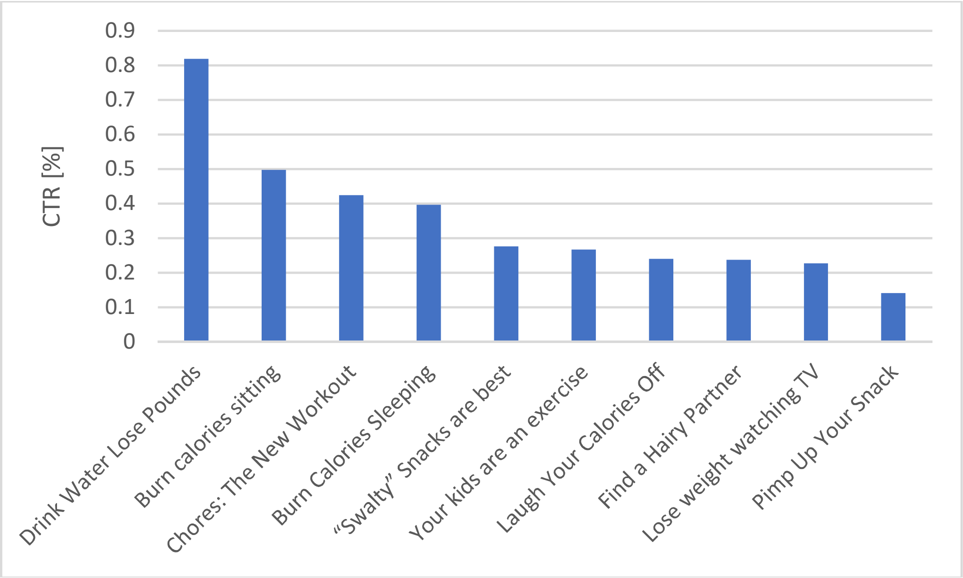

Figure S3: CTR for the different campaign ads.

Propensity score matching analysis

We used propensity score matching of users meeting inclusion characteristics, who were matched to unexposed users based on age, gender, and zip code and analyzed using the above characteristics. This analysis allows for a low-noise, low sample size analysis in which it becomes possible to obtain very conservative assurances that there are statistically significant differences by treatment status, rather than relying on clinically meaningful effect sizes (as in the parent analysis).

Comparing the treatment population with the matched controls we find that in the treatment population, 51% performed target searches after seeing the ads, compared to 41% in the matched controls (paired sign test, $P<10^{-17}$). Thus, the campaign ads increased the relative likelihood of future target searches by 24%.

A linear regression model of these population, where the independent variables are past target searches and whether the user was exposed to a campaign ad, and the dependent variable is whether the user made future target searches shows that both

variables are positively correlated and statistically significant ($P<10^{-5}$) with future target searches (R^2=0.12, $P<10^{-10}$).

Advertisements

Advertisements and their text

The text of the advertisements is shown in Table S1, and a sample advertisement in Figure S4.

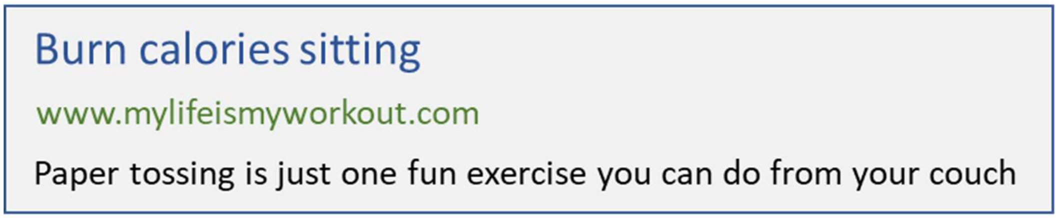

Figure S4: Sample advertisement.

Number	Title	Content
1	Burn calories sitting	Work out without leaving the comfort of the couch.
2	Burn calories sitting	Paper tossing is just one fun exercise you can do from your couch
3	Lose weight watching TV	Binge watching your favorite show can work wonders
4	Lose weight watching TV	Jumping jacks is just one easy exercise you can do during ad breaks.
5	Your kids are an exercise	The best workout time is family time.
6	Your kids are an exercise	20 minutes of frisbee or tag is a great, fun family workout.
7	“Swalty” Snacks are best	Satisfy your sweet and salty cravings at the same time
8	“Swalty” Snacks are best	Snacks that pair salty with sweet are more satisfying. Options galore!
9	Pimp Up Your Snack	Smarter snacks keep you satisfied longer.
10	Pimp Up Your Snack	Snacks that pair a protein and a good carb are yummier and better.
11	Drink Water Lose Pounds	A glass of water before a meal can work wonders
12	Drink Water Lose Pounds	A simple glass of water before a meal helps you feel fuller.
13	Burn Calories Sleeping	Turning the temperature down turns the calorie burning up.
14	Burn Calories Sleeping	Lowering the temperature is just one easy way to burn more calories.
15	Chores: The New Workout	The smallest chores can make the biggest difference.
16	Chores: The New Workout	Mopping is just one of many easy calorie-burning tasks
17	Find a Hairy Partner	Your fluffy best friend can help you burn some calories.
18	Find a Hairy Partner	Exercising with Fluffy, Fido, or Skipper keeps everyone healthy.
19	Laugh Your Calories Off	The latest workout is having a good laugh.
20	Laugh Your Calories Off	A good laugh is just one easy way to boost your metabolism.

Table S1: Title and content of the campaign ads
